# A Droplet-Based Microfluidic Platform for Rapid Optical Detection of Bacteria: Proof-of-Concept for Radiopharmaceutical Sterility Testing

**DOI:** 10.3390/mi17050532

**Published:** 2026-04-27

**Authors:** Adriano Colombelli, Daniela Lospinoso, Vita Guarino, Alessandra Zizzari, Monica Bianco, Valentina Arima, Roberto Rella, Maria Grazia Manera

**Affiliations:** 1 CNR IMM—Istituto per la Microelettronica e i Microsistemi, Campus Ecotekne, Via Monteroni, 73100 Lecce, Italy; 2 CNR Nanotec—Istituto di Nanotecnologia, Campus Ecotekne, Via Monteroni, 73100 Lecce, Italy; 3 Department of Experimental Medicine, University of Salento, Campus Ecotekne, Via Monteroni, 73100 Lecce, Italy

**Keywords:** optical sensing, microfluidics, bacteria, radiopharmaceuticals

## Abstract

Rapid sterility testing of radiopharmaceuticals is essential due to their short half-lives and strict safety requirements. Conventional culture-based methods require several days and are not compatible with clinical workflows. In this work, we present a proof-of-concept droplet-based microfluidic platform for rapid optical detection of bacterial contamination through optical extinction analysis of microdroplets. Monodisperse water-in-oil microdroplets were generated and optically interrogated using a fiber-based detection system. Calibration was first performed using 500 nm polystyrene nanoparticles to establish the relationship between particle concentration and optical extinction. Subsequently, *Staphylococcus aureus* suspensions were analyzed under aerobic and anaerobic conditions at concentrations ranging from 0 to 230 CFU/mL. The system demonstrated reliable detection of bacterial contamination with estimated limits of detection of ~15 CFU/mL (aerobic) and ~7.5 CFU/mL (anaerobic). The platform enables real-time, high-throughput analysis with minimal sample handling and reduced analysis time compared to conventional sterility tests. This study validates the feasibility of microdroplet-based optical detection as a rapid quality control strategy specifically suited for radiopharmaceutical production, where the short half-lives of common radiotracers impose strict time constraints incompatible with conventional 14-day culture-based sterility tests. The results provide a proof-of-concept foundation for future integration into automated sterility testing workflows, with further validation on real radiopharmaceutical matrices planned as the next step.

## 1. Introduction

Radiopharmaceuticals play a critical role in modern medicine, especially in diagnostic imaging and targeted therapies [1]. Their effectiveness and safety depend on stringent quality control measures to ensure purity and sterility [2]. The purity of radiopharmaceuticals refers to the absence of impurities, including chemical, radiochemical, and microbiological contaminants. Impurities can alter the radiopharmaceutical’s intended function, reduce diagnostic accuracy, and potentially lead to adverse reactions in patients [3]. Bacterial contamination is one of the most concerning threats in the production and administration of radiopharmaceuticals [4]. Contaminated preparations can cause severe infections, particularly in immunocompromised patients, who are often among those undergoing nuclear medicine checks. Septicemia and localized infections are among the possible consequences, which could compromise patient safety and lead to significant clinical complications. Maintaining sterility in the production, handling, and administration of radiopharmaceuticals requires adherence to strict Good Manufacturing Practices (GMP) and sterility testing protocols [5].

These include aseptic production environments, regular microbiological monitoring, and thorough sterilization processes. Furthermore, training personnel in aseptic techniques and enforcing rigorous quality assurance measures are vital to minimize contamination risks [5,6]. However, traditional methods of detection for impurities, particularly bacterial contamination, present several limitations that can affect their reliability and efficiency. Traditional sterility testing methods, such as culture-based techniques, require extended incubation periods often 14 days or more to confirm the absence of microbial contamination. This delay is problematic for radiopharmaceuticals, which are often used within hours or a few days due to their short half-lives [5], leading to logistical challenges and limiting timely availability for patient care [7].

While culture methods are considered the gold standard, their sensitivity depends on the growth conditions of specific microorganisms. Some fastidious or slow-growing bacteria may not be detected within the testing timeframe [7,8]. Furthermore, these methods cannot always differentiate between viable and non-viable microorganisms, which could result in inaccurate assessments. Traditional methods may miss subclinical levels of bacterial endotoxins or non-culturable microbial contaminants that remain undetectable during standard growth-based testing [9]. This poses a risk of unexpected pyrogenic reactions in patients. Moreover, chemical or radiochemical impurities are often outside the scope of conventional microbial testing methods, necessitating separate, complementary assays. Traditional sterility testing often involves manual handling, increasing the risk of human error and contamination during sample preparation. The labor-intensive nature of these methods also requires skilled personnel and extensive resources, which can pose challenges for facilities with limited expertise or budget [10]. These limitations collectively underscore the urgent need for rapid, sensitive, and automated detection technologies capable of operating within the time constraints of radiopharmaceutical production [11,12,13].

Droplet-based microfluidic systems enable the generation of monodisperse water-in-oil microdroplets through immiscible multiphase flows within microchannels, typically using T-junction or flow-focusing geometries. Each microdroplet acts as an isolated microreactor, confining reactions to pico- or nanoliter volumes and enabling high-throughput parallel processing with minimal reagent consumption [14,15]. Droplet size and generation frequency are controlled by channel geometry, fluid properties, and flow rate ratios. Stabilization against coalescence is achieved through oil-specific surfactants, ensuring chemical isolation between droplets. These features collectively enhance assay sensitivity, reduce background interference, and increase throughput compared to bulk methods [16].

Recent advances in microdroplets-based microfluidic platforms have demonstrated the potential for significantly faster detection of bacterial growth compared to traditional culture methods [14,15,17]. For instance, Kaminski et al. reported a PDMS-based microchip used to generate microdroplets containing 0–2 *E. coli* cells, enabling detection of bacterial proliferation within approximately 2 h using laser-induced fluorescence of resazurin as a metabolic indicator [18,19,20,21,22].

Alongside fluorescence-based approaches, several label-free optical methods have been developed for bacterial detection in droplet microfluidic systems, exploiting turbidity, light scattering, or absorption as detection principles. Cui et al. demonstrated smartphone-based quantification of viable bacteria through single-cell microdroplet turbidity imaging, enabling rapid and instrument-light detection of bacterial concentrations [23]. Hengoju et al. developed an optofluidic detection setup combining multiple optical parameters—including transmission and scattering—for multi-parametric analysis of microbiological samples encapsulated in droplets [24]. Kang et al. reported an ultrafast parallelized microfluidic platform capable of detecting changes in bacterial growth within 30 min at concentrations as low as 1–4 bacteria per droplet, using absorption-based readout [25]. More recently, Graf et al. demonstrated multiplexed antibiotic susceptibility testing in droplets using angle-resolved light scattering imaging, achieving rapid phenotypic profiling without any fluorescent labelling [26]. These approaches collectively confirm the analytical potential of label-free optical readout in droplet microfluidic systems, and the present work builds on this foundation by targeting the specific unmet need for rapid sterility assessment in radiopharmaceutical quality control.

The microfluidic system based on microdroplets developed by Nikolic et al. enables real-time monitoring of bacterial population dynamics within picoliter-volume droplets using time-lapse fluorescence microscopy. This system allows direct observation of bacterial growth and lysis as they occur. Unlike traditional mass or plate-reader assays, which provide averaged endpoint measurements, this approach provides continuous, high-resolution temporal data from individual droplets, capturing population- and single-cell-level behaviors in a controlled microenvironment [27,28].

Building on these advances, this work presents a proof-of-concept droplet-based microfluidic platform for rapid label-free optical detection of bacterial contamination at clinically relevant concentrations, targeting the specific unmet need for rapid sterility assessment in radiopharmaceutical quality control—an application domain where conventional culture-based testing is structurally incompatible with the short half-lives of most radiotracers. This device is proposed as a cutting-edge solution to enhance detection capabilities and streamline analytical processes. Building on these advances, this work proposes a novel approach utilizing microfluidic devices for the generation of water-in-oil microdroplets, aimed at detecting bacterial contamination in radiopharmaceutical-relevant matrices through optical extinction measurements [29,30,31]. Preliminary validation was performed using polystyrene nanospheres to assess system sensitivity to variations in light scattering associated with particle concentration, followed by biological validation using *Staphylococcus aureus* ATCC 6538 as a model organism, selected for its clinical relevance and standardized growth characteristics in sterility testing. The time constraints inherent to radiopharmaceutical production represent a particularly compelling driver for rapid sterility testing. Common diagnostic radiotracers such as ^68^Ga (t_1_/_2_ ≈ 68 min), ^18^F (t_1_/_2_ ≈ 110 min), and ^99^ᵐTc (t_1_/_2_ ≈ 6 h) decay rapidly after synthesis, leaving a narrow window for quality control before clinical administration. Conventional sterility testing, which requires up to 14 days of incubation, is therefore structurally incompatible with the radiopharmaceutical workflow: by the time a result is available, the product has long decayed. Current practice relies on retrospective testing combined with parametric release strategies, which carry an inherent residual risk. A sterility assessment method capable of delivering a result within 1–2 h would represent a substantial advancement in patient safety and operational efficiency, enabling prospective quality control within a clinically compatible time window.

In this context, this work presents a proof-of-concept microdroplet-based microfluidic platform for rapid optical detection of bacterial contamination. The system is designed to demonstrate feasibility and analytical potential, and is not intended to replace validated sterility testing protocols at this stage. Rather, it provides a proof-of-concept technological foundation for future integration into accelerated quality control workflows for radiopharmaceutical production.

## 2. Materials and Methods

### 2.1. Microfluidic Device Fabrication

Microfluidic devices were fabricated to enable precise control and manipulation of small fluid volumes, essential for applications in biomedical analysis and chemical processing. For this purpose, B-270 glass substrates coated with a 450 nm thick chromium layer were obtained from Telic. The chemical reagents used in the fabrication process, including hydrochloric acid (HCl), ammonium fluoride (NH_4_F), hydrofluoric acid (HF), and the chromium etchant, were sourced from Sigma-Aldrich (Taufkirchen, Germany).

The photolithography process employed the AZ9260 photoresist and the AZ400K developer, both supplied by MicroChemicals (Ulm, Germany). Photomasks for patterning were designed using CleWin 6 software  and printed by J. D. Photo-tools Ltd. (Oldham, Lancashire, UK). For fluidic connections, fluoropolymer tubing (Tub FEP Blu 1/32″ × 0.09″) was used, which was purchased from IDEX Health & Science (Erlangen, Germany). The flow focusing (FF) microdroplets generator was a B-270 glass-based device with an overall dimension of about 2.5 × 5 cm^2^. The microfluidic network was patterned on the glass substrate via photolithography. After geometry transfer, the glass substrate was etched with a buffered oxide etchant (BOE) solution using the microwave reactor system (Anton Paar Multiwave 3000, Labservice Analytica s.r.l., Bologna, Italy) as reported in [32], to obtain a channel depth of 100 µm and a width of 450 µm. Then, three holes were processed using a microdriller (MICROmiller MF70, Proxxon, Zweibrücken, Germany) to create two input ports and one output port. The channel was then thermally attached to a glass top plate, and finally, capillary tubes were connected to the inlet and outlet holes. After fabrication, the inner walls of the microchannels were functionalized with a hydrophobic coating. The microchannels were filled with 1H,1H,2H,2H-perfluorooctyltriethoxysilane at a constant rate of 30 µL/min using a syringe pump (Ugo Basile, Gemonio, Italy, Biological Research Apparatus, model KDS270). After complete filling, the silane was incubated for 30 min and then withdrawn at 150 µL/min. As a final step, the microchannel was dried by pumping air. This procedure ensures that the channel walls are covered by the silane layer as reported in C.M. Tone et al. [33]. After functionalization, the device was used to produce microdroplets of water in oil by injecting paraffin oil with Span80 (8 wt%) from the side channels and the aqueous solution from the central inlet. The flow rates of the continuous and dispersed phases were controlled by two independent pumps.

### 2.2. Generation of Microdroplets

The initial phase of the optical measurements on microdroplets was performed on a custom-fabricated chip, where solutions—with and without polystyrene beads—were flowed simply using a syringe pump. The droplets produced in our chip were collected in the integrated collector, which is an inherent part of the chip geometry. Due to the high stability of the droplets, it was possible to carry out optical analyses on a large number of them directly within the microfluidic collector.

The average diameter of the generated microdroplets was approximately 100 µm, as estimated from brightfield microscopy images using the calibrated scale bar. The microdroplet generation frequency was adjusted to ensure stable and reproducible optical measurements, and ranged between approximately 0.2 and 0.6 Hz depending on the experimental condition. The continuous phase consisted of paraffin oil containing 8 wt% Span80 surfactant to ensure droplet stability and prevent coalescence.

Subsequently, dynamic analyses on individual droplets were performed using a different automated microfluidic system, specifically designed to monitor each microdroplet in real time. For this purpose, microdroplets of water in oil were generated using a system that comprises a pressure controller (ElveFlow OB1 MK4), pressurized reservoirs, a microfluidic distribution valve (ElveFlow MUX), flow sensors (ElveFlow MFS), an oil reservoir, and a commercial microfluidic device capable of generating microdroplets with the same dimensions and under the same experimental conditions as the previously used system microfluidic chip. The OB1 controller ensures precise and dynamic regulation of input pressures via a feedback loop integrated with ELVEFLOW software (ESI V3.10.04). The MUX valve allows real-time selection among eight input channels, enabling microdroplets generation with diverse compositions. Flow sensors monitor and maintain stable flow rates, including for the continuous oil phase. The microfluidic chip, where microdroplets formation occurs, is integrated into the system with calibrated flow resistances to ensure reproducibility and control across experiments.

### 2.3. Experimental Set-Up for Turbidimetric Test—Calibration Procedures

The optical detection principle is based on variations in light scattering and absorbance induced by the presence and growth of bacterial cells within the microdroplets. Suspended particles and bacterial cells act as scattering centers when illuminated, producing measurable changes in the transmitted and scattered optical signal. In addition, metabolic activity in the culture medium may contribute to absorbance variations within the selected spectral range. Therefore, the detected signal reflects overall optical extinction variations associated with bacterial presence and proliferation rather than a single optical mechanism.

To calibrate and optimize the experimental setup developed in the laboratory, suitable optical extinction measurements by using optical fiber and collimator were performed on various solutions containing polystyrene nanospheres of known size (500 nm). Regarding size, many bacteria exhibit diameters or widths on the order of ~0.5–1 µm; therefore, a 500 nm polystyrene sphere can serve as a reasonable proxy for the transverse dimension of several bacterial species. For example, *Staphylococcus aureus* typically shows diameters ranging from ~0.25 to 1.0 µm. The measurements were carried out using a monochromatic light source with a wavelength of 635 nm. The purpose of this preliminary calibration was to establish an operational turbidity range and to optimize the instrumental conditions for subsequent measurements on bacterial suspensions. Although the optical characteristics of bacteria (shape, refractive index, medium absorption) differ from those of the microspheres, this calibration provides a useful qualitative reference to correlate bacterial density with turbidity, prior to establishing specific calibration curves for each strain and concentration.

The optical detection zone was positioned within the microfluidic channel using an XY translational stage on which the chip was mounted, allowing flexible and reproducible placement of the interrogation point at any location along the channel. The spectrometer used was a portable Avantes unit with an integration time of 100 ms per spectrum. The optical signal was acquired continuously as microdroplets passed through the detection zone, with each droplet generating a distinct extinction peak in the time trace.

The turbidimetric test was conducted by generating water microdroplets containing increasing concentrations of polystyrene nanoparticles with a nominal diameter of 500 nm; the particles were purchased from Sigma-Aldrich in aqueous suspensions with a concentration of 10 wt%; the coefficient of variation (CV) was specified to be 2.4% while the density of the PS particles is 1.05 g/cm^3^. Specifically, microdroplets were prepared containing pure water, as well as polystyrene nanoparticle suspensions at concentrations of 5, 10, 15 and 20 µg/mL.

**Figure 1:**
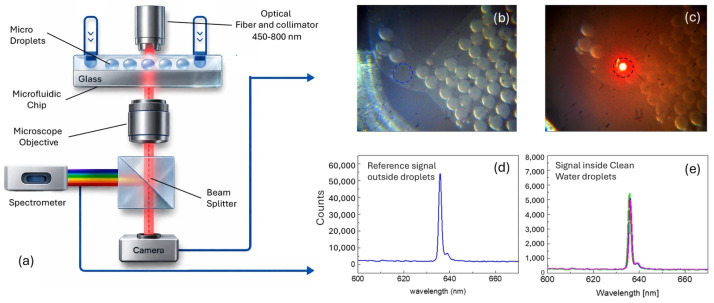
(**a**) Schematic of the optical measurement configuration. (**b**) Brightfield image of microdroplets accumulated in the collection zone of the microfluidic chip. (**c**) Image of the microdroplets during optical interrogation, showing the red laser spot (633 nm) focused on the detection region. (**d**) Reference optical signal acquired outside the microdroplets (carrier fluid only), showing the laser peak at ~633 nm. (**e**) Optical signal acquired inside clean water microdroplets, showing the attenuated laser peak used as the measurement baseline.

To monitor the variation in scattering properties of the microdroplets via turbidimetric analysis, the microdroplets were illuminated using a 633 nm red laser diode and the optical extinction signal was recorded. Figure 1 shows the experimental setup used for optical interrogation of microdroplets (panel a), representative images of the microdroplets in the collection zone with and without laser illumination (panels b,c), and the corresponding optical signals acquired outside (panel d) and inside (panel e) the microdroplets. In the final frame of this sequence, the red spot indicates the optical interrogation region inside the microfluidic channel through which the generated microdroplets pass. By tracking the scattered light signal over time, it is possible to monitor, in real time, the dynamic behavior of the microdroplets, including variations in their contents. These variations alter the optical properties of the microdroplets, which are then reflected in changes in the optical extinction signal, as depicted in the graph shown in Figure 1.

### 2.4. Sample Preparation for Bacterial Studies

Bacterial strains, comprising staphylococcus aureus ATCC 6538 species under aerobic (Tryptic Soy Broth) and anaerobic (Fluid Thioglycolate Medium) conditions, were supplied by ITEL Telecomunicazioni biolab s.r.l. 70037 Ruvo di Puglia (BA), Italy. After thawing, the suspensions were prepared in culture media at different concentrations (4.6, 9.2, 46, 92, and 230 CFU/mL). In addition, sterile control samples consisting of culture medium only (0 CFU/mL, without bacterial inoculation) were prepared in parallel under the same conditions and used as blank references for baseline establishment and LOD calculation. The prepared solutions were stored on ice to prevent bacterial proliferation and were employed for optical testing within two hours from thawing to ensure sample stability and measurement reliability.

## 3. Results

### 3.1. Turbidimetric Test Performance

Building on the microdroplet generation approach described in Section 2, calibration experiments were performed using polystyrene nanoparticles as optical standards to validate the system sensitivity prior to bacterial testing. To calibrate the microdroplets generation system, preliminary experiments were performed using microdroplets containing increasing concentrations of polystyrene nanoparticles. This choice was motivated by the scattering properties of these nanoparticles when interacting with incident monochromatic radiation. Polystyrene nanoparticles are well-suited for such studies due to their consistent size and optical properties, which cause predictable scattering behavior when exposed to light of a specific wavelength. When illuminated with monochromatic light, such as a red laser diode at a wavelength of 633 nm, these nanoparticles scatter light due to Mie scattering, which is particularly prominent for particles of sizes comparable to the wavelength of the incident light. The selection of these nanoparticles was strategic, as it allows for the simulation of the presence of biological entities, such as bacteria, cells, or other microscopic particles, within the microdroplets. This approach enables the calibration of the optical detection system using solutions with known concentrations, allowing for the precise control and interpretation of extinction data. The aim was to confirm that the system could maintain consistent microdroplets size and uniformity even in the presence of these suspended particles simulating a more complex environment, resembling real-world applications where microdroplets may contain diverse suspended materials, such as biological species or analytes. The presence of nanoparticles within the microdroplets has the potential to alter the equilibrium conditions of microdroplets formation. These particles could affect the viscosity of the solution, as well as the interfacial tension between the aqueous microdroplets and the surrounding continuous phase, leading to variations in microdroplets size, shape, or production speed. Higher concentrations of nanoparticles might increase the internal viscosity of the microdroplets, potentially slowing down the microdroplets formation process or leading to less uniform microdroplets sizes. However, through careful adjustment of flow rates and pressure in the microfluidic channels, it was possible to mitigate these effects.

**Figure 2:**
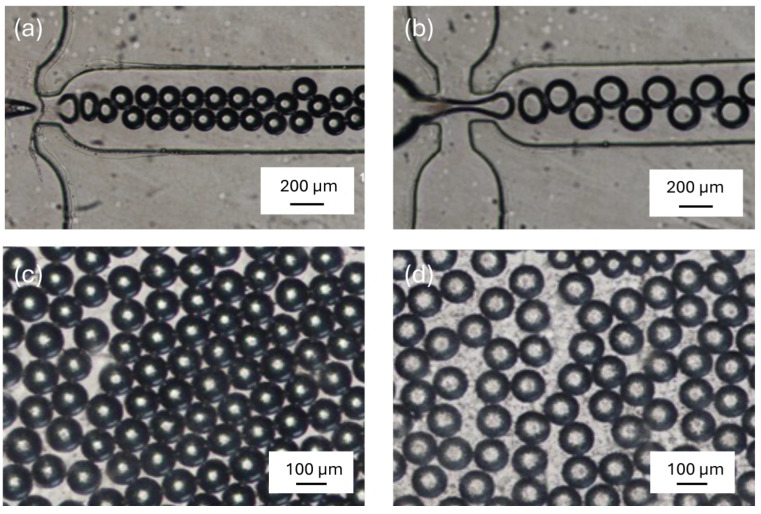
Microscopy images of microdroplet generation and accumulation in the microfluidic device. (**a**) Brightfield image of microdroplets forming at the flow-focusing junction during generation (scale bar: 200 µm). (**b**) Brightfield image of larger microdroplets at the junction under different flow conditions (scale bar: 200 µm). (**c**) Microdroplets accumulated in the collection zone without polystyrene nanoparticles (clean droplets), showing uniform size distribution (scale bar: 100 µm). (**d**) Microdroplets accumulated in the collection zone containing polystyrene nanoparticles, showing increased optical contrast compared to clean droplets (scale bar: 100 µm).

The microdroplets generation system demonstrated the ability to produce microdroplets of uniform size even as the concentration of polystyrene nanoparticles increased. Consistent microdroplets diameters, as shown in Figure 2 (panels a,b), confirmed that the system could adapt to these variations without compromising the homogeneity of microdroplets size or the regularity of microdroplets production. This outcome is crucial for ensuring that subsequent optical measurements remain consistent and that any changes in detected signals are attributable to variations in analyte concentration rather than inconsistencies in microdroplets formation. As shown in Figure 2 (panels c,d), a clear difference in optical contrast is observable between clean microdroplets and those containing polystyrene nanoparticles.

**Figure 3:**
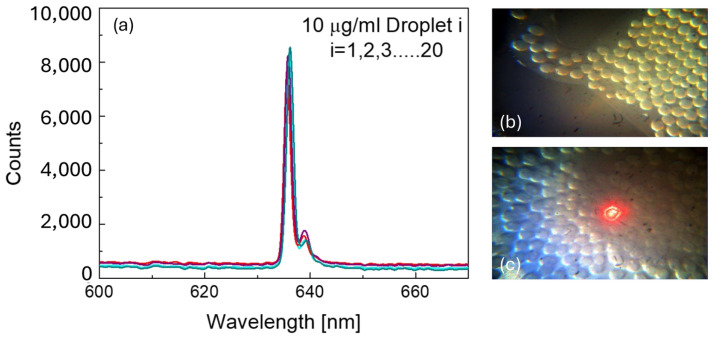
Optical reproducibility of the measurement system. (**a**) Overlay of optical extinction spectra acquired from n = 20 individual microdroplets (i = 1, 2, 3, …, 20) containing 10 µg/mL polystyrene nanoparticles, demonstrating high signal reproducibility across droplets. (**b**) Brightfield image of microdroplets in the collection zone under white light illumination. (**c**) Image of microdroplets during laser interrogation, showing the red spot at the optical detection point.

Moving towards the calibration of the experimental set-up, Figure 3 puts in evidence optical extinction corresponding to microdroplets containing a concentration of 10 µg/mL of polystyrene nanoparticles. This visual comparison highlights the effect of the nanoparticle concentration on the optical properties of the microdroplets, providing further confirmation of the system’s ability to produce uniform microdroplets under varying internal conditions. The spectrum shown in Figure 3 highlights the counts acquired by the spectrophotometer as the optical beam passes through individual microdroplets. These counts represent a measure of the extinction (scattering and absorption) associated with each investigated microdroplets. To confirm the reproducibility of the measurement, at least 20 microdroplets from the accumulation zone were selected and analyzed for each tested concentration.

**Figure 4:**
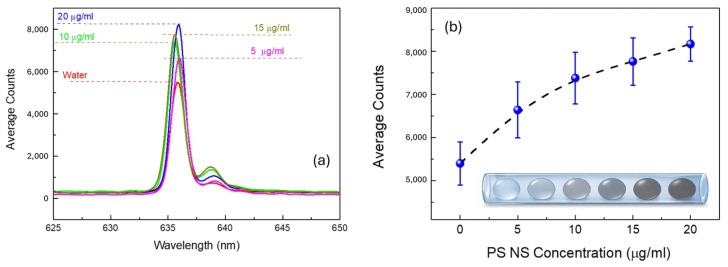
Calibration of the optical detection system using polystyrene nanospheres (PS NS). (**a**) Averaged optical extinction spectra obtained from n = 20 microdroplets per concentration, for pure water and PS NS suspensions at 5, 10, 15, and 20 µg/mL. (**b**) Calibration curve showing average peak counts as a function of PS NS concentration (µg/mL), with error bars representing the standard deviation (SD, n = 20). The inset illustrates the progressive increase in microdroplet turbidity with increasing nanoparticle concentration. Statistical significance between the 20 µg/mL group and all lower concentration groups was confirmed by one-way ANOVA with Tukey’s post-hoc test (*p* < 0.05).

The graph in Figure 4 further quantifies these observations by showing the scattering intensity, as in the previous situation, for microdroplets containing pure water (red line), 5, 10, 15 and 20 µg/mL of nanoparticles. These curves represent the averaged scattering signal obtained from measurements of 20 individual microdroplets for each nanoparticles concentration, ensuring statistical reliability and minimizing variability in the data.

As can be noticed also in the calibration curve reported in Figure 4, the intensity of scattered light increases with the concentration of nanoparticles, as expected. This trend is consistent with the principles of Mie scattering, where the scattering intensity is proportional to the concentration and size of the scattering centers in this case, the polystyrene nanoparticles.

Notably, the peak intensity for the 20 µg/mL sample is significantly higher than that of the 5, 10, and 15 µg/mL and water-only samples (one-way ANOVA followed by Tukey’s post-hoc test, *p* < 0.05, n = 20 microdroplets per group), demonstrating the system’s ability to distinguish different levels of particle concentration. The broader peak in the 20 µg/mL sample also suggests that the increased nanoparticle concentration contributes to a more significant scattering effect, leading to higher overall turbidity. This result confirms that the optical detection system is sensitive enough to differentiate between varying levels of suspended particles within the microdroplets. The sharp rise and fall in the scattering intensity over time indicate uniform microdroplets sizes and consistent behavior during their passage through the optical interrogation region. This consistency is crucial for ensuring reliable measurements in subsequent applications, such as miniaturized bacteria growth tests, where precise detection of small variations is required.

The differences observed between the microdroplets with only water and those containing nanoparticles validate the capability of the microfluidic system to maintain homogeneous microdroplets generation even when particulate matter is introduced. This outcome is particularly important for ensuring that the optical measurements accurately reflect the concentration of nanoparticles rather than inconsistencies in microdroplets size or shape. This capability lays the groundwork for further applications in biosensing and other microfluidic assays, where precise control over microdroplets contents and their optical properties is essential.

### 3.2. Calibration Strategy Based on Simulated Bacterial Growth

Based on the previous validation with synthetic standards, we subsequently investigated bacterial suspensions under both aerobic and anaerobic conditions, in order to assess the capability of the system to detect microbial growth at clinically relevant concentrations. The results obtained in this study confirm the feasibility of the proposed approach. It is important to emphasize that this work presents a proof-of-concept platform demonstrating rapid optical detection capability within microdroplets. The objective is to validate the analytical principle rather than to provide a fully validated clinical sterility testing system.

The calibration of the optical system was first performed using 500 nm polystyrene spheres illuminated at 633 nm. This wavelength was specifically selected to exploit Mie scattering in a spectral region free from absorption by the culture media, ensuring that the detected signal reflects particle-induced scattering only. This approach allowed us to validate the system response to increasing particle concentrations under well-controlled conditions, confirming both linearity and reproducibility of the extinction signal. While these synthetic standards provide a reliable benchmark, it is important to note that bacterial cells differ in several respects: they exhibit irregular morphology, a broader size distribution, and a refractive index closer to that of the culture medium [34]. For these reasons, the calibration curve obtained with polystyrene spheres cannot be directly transferred to bacterial solutions, and separate calibration curves were constructed for each bacterial condition (aerobic and anaerobic) using the respective culture media.

**Figure 5:**
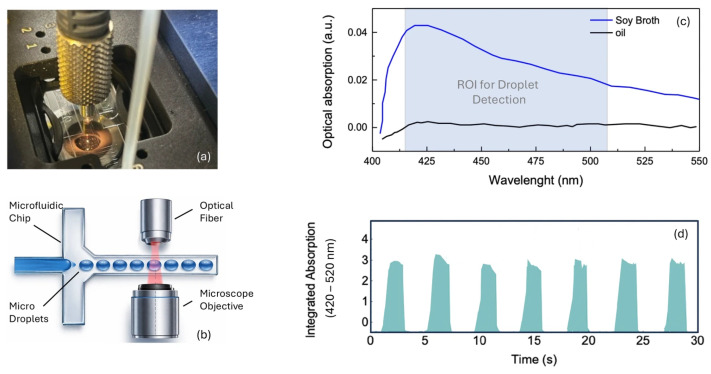
Optical characterization of the bacterial detection system using culture medium microdroplets. (**a**) Photograph of the automated microfluidic system showing the chip holder and optical fiber connection. (**b**) Schematic of the optical detection configuration for bacterial measurements, comprising the microfluidic chip, optical fiber, and microscope objective. (**c**) Absorbance spectra of sterile Tryptic Soy Broth (Soy Broth, blue) and paraffin oil (black) microdroplets, highlighting the region of interest (ROI) for droplet detection in the 420–510 nm range. (**d**) Representative time trace of the integrated optical absorption (420–520 nm) acquired from sterile culture medium microdroplets passing through the detection zone, used as the baseline reference for bacterial measurements.

For bacterial detection, the optical regime was intentionally different from that used for PS calibration. A broadband white LED source was employed and the integrated absorbance in the 420–520 nm range was monitored, because the culture media (Tryptic Soy Broth for aerobic conditions, Fluid Thioglycolate Medium for anaerobic conditions) exhibit characteristic optical absorption in this spectral window, as shown in Figure 5c. This absorption correlates with the optical properties of the medium and its modification by bacterial metabolic activity and growth. Additional contributions from absorption by cellular components are also expected in this range. The two optical regimes are therefore complementary rather than inconsistent: the 633 nm PS calibration establishes system sensitivity and linearity under controlled scattering conditions, while the 420–520 nm LED-based measurement provides the actual bacterial detection signal in conditions that closely mimic the sterility testing matrix. Nevertheless, the use of polystyrene particles was essential to establish a robust baseline and to demonstrate the sensitivity of the optical setup to small variations in turbidity, ensuring that subsequent bacterial measurements can be reliably interpreted as arising from microbial presence rather than instrumental variability.

Once the measurement system was calibrated, tests were conducted in the presence of bacterial loads at known concentrations, with the aim of constructing calibration curves suitable for both aerobic and anaerobic bacterial populations. To achieve this, a specific culture medium was used as the carrier liquid, selected according to the type of samples to be analyzed. The range of concentrations tested included sterile control microdroplets (0 CFU/mL, culture medium without bacterial inoculation), which served as the blank reference for baseline signal determination and LOD calculation, followed by progressively increasing bacterial loads: 4.6, 9.2, 46, 92, and 230 CFU/mL. The sterile controls were prepared and measured under identical conditions to the inoculated samples, ensuring a direct and meaningful comparison. Bacterial concentrations in the indicated range are sufficiently low to simulate accidental or minimal contamination in sterile products. This is crucial for assessing the system’s ability to detect even small amounts of contaminants, thereby ensuring the reliability of the sterilization process [35].

As shown in Figure 5, the automated detection setup (panels a,b) was employed for bacterial measurements. The culture medium exhibits characteristic optical absorption in the 420–510 nm range (panel c), clearly distinguishable from the oil signal, providing the spectral basis for bacterial detection. Panel (d) shows the time-resolved integrated absorption signal from sterile medium microdroplets used as baseline reference.

**Figure 6:**
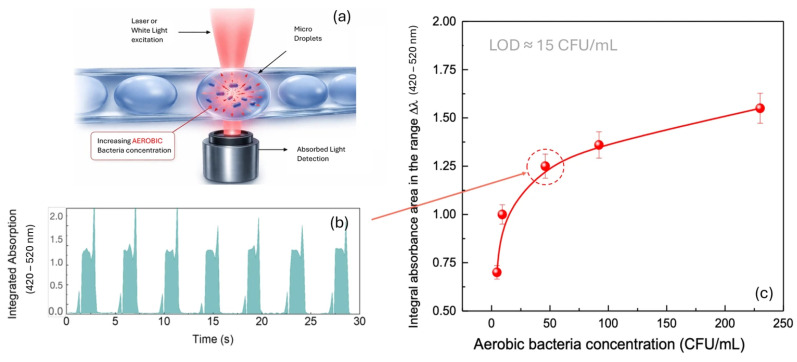
Optical detection of aerobic bacterial contamination. (**a**) Schematic of the detection principle: laser or white light excitation of microdroplets containing increasing concentrations of aerobic *Staphylococcus aureus* ATCC 6538, with absorbed light collected by the detector below. (**b**) Representative time trace of integrated optical absorption (420–520 nm) from microdroplets containing aerobic bacteria at a known concentration. (**c**) Calibration curve showing the integral absorbance area in the range Δλ (420–520 nm) as a function of aerobic bacterial concentration (CFU/mL), with error bars (SD, n = 20).

Figure 6 illustrates the optical detection of aerobic bacterial contamination. Panel (a) shows the schematic of the detection principle, in which microdroplets containing increasing concentrations of aerobic *Staphylococcus aureus* ATCC 6538 are interrogated by laser or white light excitation and the absorbed light is collected below. Panel (b) shows a representative time trace of the integrated optical absorption (420–520 nm) acquired from microdroplets at a known aerobic bacterial concentration, where each peak corresponds to a single microdroplet passing through the detection zone. Panel (c) shows the corresponding calibration curve of the integral absorbance area in the range Δλ (420–520 nm) as a function of aerobic bacterial concentration (CFU/mL). The optical response increases with bacterial concentration, showing a clear trend up to approximately 92 CFU/mL. The sensitivity is most pronounced in the low concentration range (0–9.2 CFU/mL), which is critical for early contamination detection. An estimated limit of detection (LOD) of approximately 15 CFU/mL was obtained from the first linear region of the curve using the 3σ/S criterion, indicating that the system can effectively detect early-stage bacterial presence, though further optimization may be required to enhance resolution near the detection threshold.

**Figure 7:**
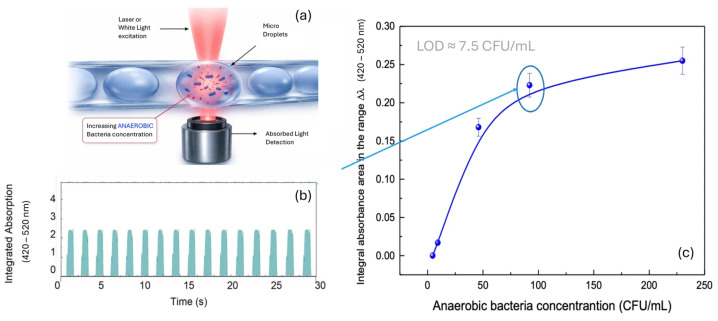
Optical detection of anaerobic bacterial contamination. (**a**) Schematic of the detection principle: laser or white light excitation of microdroplets containing increasing concentrations of anaerobic *Staphylococcus aureus* ATCC 6538 in Fluid Thioglycolate Medium. (**b**) Representative time trace of integrated optical absorption (420–520 nm) from microdroplets containing anaerobic bacteria at a known concentration, showing uniform droplet signals. (**c**) Calibration curve showing the integral absorbance area in the range Δλ (420–520 nm) as a function of anaerobic bacterial concentration (CFU/mL), with error bars (SD, n = 20).

Figure 7 presents the optical detection of anaerobic bacterial contamination. Panel (a) shows the schematic of the detection principle, analogous to the aerobic case, in which microdroplets containing increasing concentrations of anaerobic *Staphylococcus aureus* ATCC 6538 in Fluid Thioglycolate Medium are optically interrogated and the absorbed light collected below. Panel (b) shows a representative time trace of the integrated optical absorption (420–520 nm) from anaerobic bacterial microdroplets, where the uniform peak height and spacing confirm the stability and reproducibility of droplet generation under anaerobic culture conditions. Panel (c) shows the calibration curve of the integral absorbance area in the range Δλ (420–520 nm) as a function of anaerobic bacterial concentration (CFU/mL). The trend follows the same behavior observed under aerobic conditions, with an initial linear increase and eventual saturation at higher concentrations. The sensitivity in the lower concentration range confirms the system’s effectiveness in detecting minimal bacterial presence. An estimated limit of detection (LOD) of approximately 7.5 CFU/mL was obtained from the first linear region using the 3σ/S criterion, highlighting an even higher sensitivity compared to aerobic measurements and confirming the potential of the optical method for early contamination monitoring in anaerobic environments.

The results obtained from the calibration curves for both aerobic and anaerobic bacterial populations demonstrate the system’s capability to detect low bacterial concentrations in a rapid and reproducible manner. In both cases, the optical response shows a consistent increase with increasing CFU/mL, validating the sensitivity of the turbidimetric approach in the microdroplets format. The saturation observed at higher bacterial concentrations indicates a limit in linearity beyond 46–92 CFU/mL, suggesting that the most accurate quantification range lies within the lower concentration interval (0–9.2 CFU/mL), which is particularly relevant for sterility testing where early contamination must be identified promptly.

The reproducibility of the optical signal across multiple microdroplets confirms the stability of microdroplets generation and the robustness of the optical setup. This ensures that observed variations in absorbance are predominantly due to changes in bacterial concentration rather than technical variability.

Importantly, the use of culture medium within microdroplets mimics realistic sample conditions encountered in quality control workflows, further supporting the applicability of this system for practical sterility assessments.

## 4. Discussion

Compared to representative state-of-the-art droplet-based bacterial detection approaches, the proposed platform offers a competitive combination of speed, sensitivity, and simplicity, as summarized in Table 1. Kang et al. demonstrated single-bacterium detection in unprocessed blood using the Integrated Comprehensive Droplet Digital Detection (IC3D) system, achieving a limit of detection of approximately 1 CFU/mL within ~4 h; however, this approach relies on fluorescence labelling and dedicated optical infrastructure [17]. Similarly, Nikolic et al. employed time-lapse fluorescence microscopy within picoliter droplets to monitor bacterial population dynamics at the single-cell level, providing high temporal resolution but requiring fluorescent reporters and extended acquisition times [21]. Ge et al. reported a label-free droplet-based platform for bacterial growth phenotype screening with a LOD of ~10 CFU/mL in approximately 3 h, representing a closer analogy to the present work in terms of detection principle [29].

More recently, Agnihotri et al. demonstrated a droplet microfluidics approach for the detection of rare antibiotic-resistant subpopulations of *E. coli* from bloodstream infections, using label-free observation of droplet shrinkage under microscopy [36]. While this approach targets antibiotic susceptibility testing rather than sterility assessment, it further confirms the versatility of droplet-based platforms for label-free bacterial analysis at clinically relevant concentrations.

In contrast, conventional culture-based sterility testing, while remaining the regulatory gold standard, requires up to 14 days to confirm the absence of contamination—a timeline incompatible with the short half-lives of most radiopharmaceuticals. The platform presented here achieves estimated LODs of ~15 CFU/mL (aerobic) and ~7.5 CFU/mL (anaerobic) within less than 2 h, without requiring fluorescent labels, metabolic reporters, or complex optical setups. These characteristics make it particularly suited for time-constrained quality control workflows in radiopharmaceutical production.

The lower limit of detection observed under anaerobic conditions compared to aerobic conditions can be primarily attributed to the different optical baselines of the two culture media employed. Fluid Thioglycolate Medium (FTM), used for anaerobic testing, contains reducing agents—including sodium thioglycolate and L-cystine—that exhibit characteristic absorption in the 420–520 nm detection range, resulting in a higher baseline absorbance signal compared to Tryptic Soy Broth (TSB). This elevated baseline increases the overall signal-to-noise ratio for detecting small optical extinction changes induced by bacterial presence at low concentrations, effectively improving sensitivity and lowering the apparent LOD under anaerobic conditions. Additionally, *Staphylococcus aureus* is a facultative anaerobe and may exhibit differences in cell morphology and membrane composition depending on growth conditions, which could further contribute to differences in light scattering and absorption properties between the two experimental conditions.

Potential sources of signal variability—including droplet instability, coalescence, and evaporation—were carefully evaluated. Droplet stability was ensured throughout the measurements by the use of Span80 surfactant at 8 wt% in the continuous paraffin oil phase, which effectively prevents coalescence by reducing interfacial tension. Droplet size uniformity was monitored throughout each experiment and maintained within a coefficient of variation below 5%, confirming consistent droplet generation. No coalescence events were observed during the measurement window. Evaporation was considered negligible given the short total acquisition time (<2 h from droplet generation) and the sealing effect of the surrounding oil phase. These observations confirm that the variations in the optical signal are predominantly attributable to differences in bacterial concentration rather than to artefacts introduced by droplet instability.

The compatibility of the proposed platform with radiopharmaceutical workflows requires careful consideration. With a total analysis time currently estimated at less than 2 h, the platform is most directly applicable to ^99^ᵐTc-labelled compounds (t_1_/_2_ ≈ 6 h), which represent the most widely used class of diagnostic radiotracers and for which a pre-release sterility signal could in principle be obtained within a clinically compatible time window. For shorter-lived isotopes such as ^68^Ga (t_1_/_2_ ≈ 68 min) and ^18^F (t_1_/_2_ ≈ 110 min), further optimization of the sample handling and measurement workflow would be required, or the analysis would need to be run in parallel with synthesis and other quality control procedures within an automated GMP-compliant setup.

A critical next step toward clinical translation is the validation of the platform in real radiopharmaceutical matrices. Unlike standard culture media, radiopharmaceutical solutions may contain chelating agents (e.g., EDTA, DTPA), pH buffers, stabilizers, and radiolytic degradation products that could contribute to background optical absorption in the 420–520 nm detection range, potentially affecting the signal-to-noise ratio and the LOD. Dedicated matrix-matched calibration curves, blank correction strategies, and interference studies will be required before the system can be applied to actual radiopharmaceutical quality control. Additionally, a rigorous quantification of detection time as a function of bacterial concentration—with replicated measurements and associated standard deviations—is necessary to provide a statistically supported estimate of time-to-detection and to fully characterize the platform’s compatibility with specific radiopharmaceutical half-lives.

The present results establish the feasibility of rapid label-free optical detection in microdroplets at clinically relevant contamination levels, providing the analytical foundation for the translational steps outlined above.

## 5. Conclusions

This study demonstrates the successful development of a microfluidic device capable of generating and analyzing optically distinguishable microdroplets for rapid bacterial detection, with shorter analysis times compared to traditional sterility tests. The system achieved estimated LODs of ~15 CFU/mL (aerobic) and ~7.5 CFU/mL (anaerobic), calculated via the 3σ/S criterion, within a detection time of less than 2 h. From a radiopharmaceutical perspective, the sub-2 h detection time demonstrated here is particularly relevant. Unlike conventional culture-based sterility tests, which return results only after the product has already been administered or discarded, the proposed platform could in principle provide a sterility signal within the operational half-life of several common radiotracers, supporting a shift from retrospective to prospective quality control. Although validation on real radiopharmaceutical matrices, mixed microbial populations, and under GMP-compliant conditions will be required before any clinical implementation, this proof-of-concept study establishes the technical and analytical foundation for such a development. The use of microdroplets microfluidics combined with optical sensing measurements allowed for rapid calibration and detection of bacterial concentrations as low as 4.6 CFU/mL, providing better sensitivity compared to standard methods such as culture-based assays, which require significantly longer processing times. In particular, optical sensing was based on the compounds released by the few bacteria present in the medium and was evaluated via absorbance in the 420–520 nm range. Moreover, under aerobic and anaerobic conditions, bacteria may produce different metabolites or in varying amounts, which could also explain the observed differences in the limit of detection (LOD) for the same bacterial species. The effectiveness of the method lies in its ability to maintain microdroplets uniformity and optical measurement stability, ensuring that variations in the acquired signal are directly attributable to changes in bacterial concentration. The interpretation of results is straightforward, as the increase in optical extinction correlates with bacterial growth, both in aerobic and anaerobic conditions, enabling reliable and quantitative analysis in real time. Possible improvements of the system include the integration of additional sensing modalities, such as fluorescence-based detection or electrical impedance, which could enhance specificity and enable the simultaneous detection of multiple biological parameters. Moreover, further automation of the analytical workflow, including microdroplets generation, detection, and data analysis, could lead to a fully integrated and user-friendly diagnostic platform. Although promising, the present platform is not intended to replace validated sterility testing methods at this stage. Further studies involving real radiopharmaceutical matrices, mixed microbial populations, regulatory validation protocols, and interlaboratory testing will be required before clinical implementation. Nevertheless, this proof-of-concept study establishes the technical feasibility of rapid microdroplet-based optical sterility assessment. From a practical standpoint, this technology holds considerable promise for application in radiopharmaceutical quality control, particularly for ^99^ᵐTc-labelled compounds where the 6 h half-life provides a sufficiently wide window for pre-release sterility assessment. However, several critical steps remain before clinical implementation can be considered. These include: (i) validation of the detection system in real radiopharmaceutical matrices, with assessment of potential optical interferences from matrix components; (ii) rigorous quantification of time-to-detection with associated statistical uncertainty across bacterial concentration ranges; (iii) testing with mixed microbial populations and other pharmacopeial test organisms; and (iv) regulatory validation under GMP-compliant conditions. The present proof-of-concept study establishes the analytical foundation for this translational pathway, demonstrating the feasibility of the optical detection principle at clinically relevant contamination levels.

## Figures and Tables

**Table 1:** Comparison of the proposed microdroplet-based optical platform with representative state-of-the-art bacterial detection methods.

Method/Reference	Detection Time	LOD (CFU/mL)	Technique	Key Advantage
This work (microdroplet + turbidimetry)	<2 h	~7.5 (anaerobic)/~15 (aerobic)	Optical scattering/absorbance	Label-free, no incubation, real-time
Kang et al., Nat. Commun. 2014 [17]	~4 h	~1	Fluorescence (IC3D)	Single-bacterium sensitivity
Nikolic et al., Front. Microbiol. 2023 [21]	~2–4 h	single-cell	Time-lapse fluorescence microscopy	Population dynamics monitoring
Ge et al., Sens. Actuators B 2023 [25]	~3 h	~10	Label-free optical/growth phenotype	No labelling required
Agnihotri et al., 2025 [36]	~4–6 h	Subpopulation level	Droplet shrinkage (label-free microscopy)	Detects rare resistant subpopulations
Standard culture-based sterility test (Ph. Eur. 2.6.1)	14 days	1–10	Colony growth	Regulatory gold standard

## Data Availability

The original contributions presented in this study are included in the article. Further inquiries can be directed to the corresponding authors.
